# Characterization of Near Full-Length Transmitted/Founder HIV-1 Subtype D and A/D Recombinant Genomes in a Heterosexual Ugandan Population (2006–2011)

**DOI:** 10.3390/v14020334

**Published:** 2022-02-07

**Authors:** Sheila N. Balinda, Anne Kapaata, Rui Xu, Maria G. Salazar, Allison T. Mezzell, Qianhong Qin, Kimberly Herard, Dario Dilernia, Anatoli Kamali, Eugene Ruzagira, Freddie M. Kibengo, Heeyah Song, Christina Ochsenbauer, Jesus F. Salazar-Gonzalez, Jill Gilmour, Eric Hunter, Ling Yue, Pontiano Kaleebu

**Affiliations:** 1 Medical Research Council, UVRI & LSTHM Uganda Research Unit, Plot 51–59, Entebbe, Uganda; Anne.Kapaata@mrcuganda.org (A.K.); lupita.g.salazar12@gmail.com (M.G.S.); Eugene.Ruzagira@mrcuganda.org (E.R.); Freddie.Kibengo@mrcuganda.org (F.M.K.); j.sal.gonz.3@gmail.com (J.F.S.-G.); Pontiano.Kaleebu@mrcuganda.org (P.K.); 2 Emory Vaccine Center, Yerkes National Primate Research Center, Atlanta, GA 30329, USA; rui.xu2@emory.edu (R.X.); qianhong.qin@emory.edu (Q.Q.); kherard3@gmail.com (K.H.); ddilern@emory.edu (D.D.); hjohn24@emory.edu (H.S.); ehunte4@emory.edu (E.H.); lyue2@emory.edu (L.Y.); 3 Department of Molecular Genetics, Biochemistry, and Microbiology, University of Cincinnati College of Medicine, 3230, Eden Ave, Cincinnati, OH 45267, USA; mezzelat@mail.uc.edu; 4 International AIDS Vaccine Initiative (IAVI), Nairobi 00202, Kenya; AKamali@iavi.org; 5 Department of Medicine, University of Alabama at Birmingham, Birmingham, AL 35294, USA; christinaochsenbauer@uabmc.edu; 6 International AIDS Vaccine Initiative (IAVI), Imperial College London, London SW10 9NH, UK; jill_gilmour@hotmail.co.uk; 7 Department of Pathology and Laboratory Medicine, Emory University, Atlanta, GA 30329, USA

**Keywords:** HIV-1, subtype D, recombinant, T/F

## Abstract

Detailed characterization of transmitted HIV-1 variants in Uganda is fundamentally important to inform vaccine design, yet studies on the transmitted full-length strains of subtype D viruses are limited. Here, we amplified single genomes and characterized viruses, some of which were previously classified as subtype D by sub-genomic pol sequencing that were transmitted in Uganda between December 2006 to June 2011. Analysis of 5′ and 3′ half genome sequences showed 73% (19/26) of infections involved single virus transmissions, whereas 27% (7/26) of infections involved multiple variant transmissions based on predictions of a model of random virus evolution. Subtype analysis of inferred transmitted/founder viruses showed a high transmission rate of inter-subtype recombinants (69%, 20/29) involving mainly A1/D, while pure subtype D variants accounted for one-third of infections (31%, 9/29). Recombination patterns included a predominance of subtype D in the *gag*/*pol* region and a highly recombinogenic envelope gene. The signal peptide-C1 region and gp41 transmembrane domain (Tat2/Rev2 flanking region) were hotspots for A1/D recombination events. Analysis of a panel of 14 transmitted/founder molecular clones showed no difference in replication capacity between subtype D viruses (*n* = 3) and inter-subtype mosaic recombinants (*n* = 11). However, individuals infected with high replication capacity viruses had a faster CD4 T cell loss. The high transmission rate of unique inter-subtype recombinants is striking and emphasizes the extraordinary challenge for vaccine design and, in particular, for the highly variable and recombinogenic envelope gene, which is targeted by rational designs aimed to elicit broadly neutralizing antibodies.

## 1. Introduction

According to the Joint United Nations Programme on HIV/AIDS (UNAIDS)report, an estimated 2 million individuals are newly infected with HIV-1 every year [1]. Phylogenetic analyses of HIV-1 strains have revealed four major groups: M, N, O and P. Group M is responsible for the majority of infections and is sub-divided into nine subtypes A, B, C, D, F, G, H, J and K. Recently, subtype L has been identified in Central Africa and is yet to be confirmed (Yamaguchi et al., 2020).

Globally, it is estimated that approximately 20% of new HIV infections are due to HIV-1 circulating recombinant forms (CRFs) and unique recombinant forms (URFs), particularly in regions such as Africa, where several subtypes are known to co-circulate [2]. Previous studies investigating HIV-1 diversity in Uganda have targeted sub-genomic fragments: *gag*, [3] *pol* [4,5] and *env* [3]. In addition, full-length genomes in other studies were performed mainly in chronically infected individuals in rural and semi-urban areas, focusing on the southern and central regions of Uganda. In these studies, inter-subtype recombinant viruses (mostly A-D URFs) were estimated to occur in a range of 6–19% of total infections based on sub-genomic data [3,5,6,7] and nearly 30% based on full-length genome data [8]. As sequence diversity assessments are largely based on the *gag-pol* and env genes instead of the full HIV-1 genome, it is plausible that current figures of the frequency of HIV recombinants are an underestimate. More recent estimates using near full-length genome data from the same geographic regions in Uganda showed that 46% and 49.9% of the 200 and 465 patients, respectively, were infected by recombinant viruses, with A1D recombinants being most frequently observed (25%) [9,10].

In approximately 80% of heterosexual transmission events of HIV-1, a single virus, the transmitted/founder (T/F) virus, is thought to be transmitted and establish infection in the naive host from the diverse quasi-species in the chronically infected donor [11,12]. Understanding the key features of T/F viruses further provides additional insights into mechanisms underlying transmission, which is important for both vaccine design and therapeutic interventions [13]. Most T/F studies have used subtype B and C viruses to determine the phenotypic characteristics relative to the chronic viruses. Both Deymier et al. and Iyer et al. studied genomes and clones from six and eight epidemiologically linked transmission pairs, respectively [13,14]. Deymier et al. observed that T/Fs from subtype C showed little difference in replicative capacity and resistance to interferon alpha compared to non-transmitted variants within each transmission pair. In contrast, Iyer et al.’s data demonstrated that T/F are relatively more resistant to both interferon alpha and beta relative to the chronic viruses for both subtype B and C strains [13,14]. The difference in data could be attributed to the different sample types used, female–male transmissions compared to male–female transmissions and the subtypes studied. Baalwa et al., 2013 characterized 12 T/F viruses of subtypes A, D and A/D and discovered that all 12 viruses used CCR5 but not CXCR4 as a co-receptor. Additionally, T/F of subtype D replicated more efficiently compared to subtype A viruses assayed in primary human CD4+ T cells [15].

As there have been limited studies on subtype D T/F viruses, the current study focused on near full-length genome analyses of viruses previously classified, based on the pol gene. In addition, we investigated features of HIV-1 infections and patient outcomes related to the full genome sequence and in vitro replication of T/F viruses.

## 2. Materials and Methods

### 2.1. Study Subjects

The International AIDS Vaccine Initiative (IAVI) Protocol C acute HIV infection cohort was a prospective multi-center observational study that enrolled approximately 600 volunteers 18–60 years of age with recent HIV infection [16]. Study sites were in Kenya, Uganda, Rwanda, Zambia and South Africa.

Newly infected individuals were identified through p24 ELISA and serological tests and followed up at regular intervals for up to 8 years. All volunteers received HIV care, including Antiretroviral Therapy (ART) according to national guidelines. The estimated date of infection (EDI; Table 1) was defined as either (i) the midpoint between the last negative and first positive HIV antibody test, (ii) 14 days before the first positive p24 antigen test, (iii) 10 days before the first positive HIV-1 viral load test in the absence a positive p24 antigen or rapid HIV antibody result or (iv) the date of a self-reported high-risk exposure event. Additionally, we used the Poisson fitter tool and compared the time to most recent common ancestor (TMRCA) derived from 5′ and 3′ half genome sequences from a set of 20 study volunteers with single virus transmissions.

**Figure 1:**
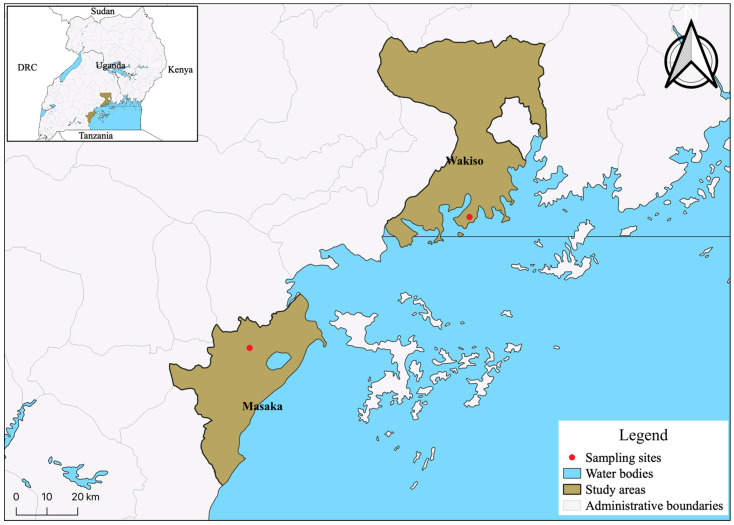
The study area in Masaka, a rural setting in the southern region of the country, and Entebbe, an urban setting in the Wakiso District in central Uganda, are labelled as red dots on the map. The image was rendered using QGIS.

The cohort is ideal for studies of both very early infection HIV isolates as well as immunologic and viral markers of HIV disease progression. In Uganda, we focused on acutely HIV-infected participants from two sites: Masaka, a rural district in south-western Uganda, and Entebbe, an urban area in Wakiso district, central Uganda (Figure 1). The overall subtype distribution for sero-converters during the study period (December 2006 to June 2011) at the two sites was 50% subtype D, 38% A1, 3.5% C and 8.5% recombinant based on pol sequencing.

### 2.2. HIV-1 Near Full-Length and Half Genomes

#### 2.2.1. Amplification of Near Full-Length Genomes

In-house primers used in this study and their details are listed in Table 1. Viral RNA was extracted from plasma and full-length cDNA synthesized as described previously [17]. Briefly, 140 µL of patient plasma was used to extract viral RNA using the QIAamp Viral RNA Mini Kit (Qiagen Inc, Valencia, CA, USA). RNA was recovered and used to synthesize near full-length HIV cDNA using SuperScript III Reverse Transcriptase (Life Technologies, USA: Invitrogen, Ljubljana, Slovenia) enzyme with primers 1′3′3′ and OFM19 (Table 1). Near full-length (NFL) cDNA was serially diluted in replicates of eight PCR wells and subjected to nested PCR amplification with HIV-specific primers (Table 1) to yield ~9-kb amplicon at a dilution in which 30% of the wells tested positive for amplification [18]. In a total reaction volume of 25 µL, 1X Q5 Reaction Buffer, 1X Q5 High GC Enhancer, 0.35 mM of each dNTP, 0.5 M of primers 1.U5Cc and 1.3′3′PlCb and 0.02 U/mL of Q5 Hot Start High-Fidelity DNA Polymerase (NEB) were used for the first round of PCR. PCR conditions for the first round were: 98 °C for 30 s, followed by 30 cycles of 98 °C for 10 s, 72 °C for 7.5 min, with a final extension of 72 °C for 10 min. One microliter of first-round PCR product was then used as a template for the second-round PCR, with identical cycling conditions and PCR mix except for the primers, whereby primer 2.U5Cd and 2.33plCb were used. PCR reactions were then run at 300 V for 25 min on a 1 percent agarose lithium acetate gel to detect the presence of a ~9 kb band. For samples that did not amplify using the ~9 kb fragment, a half genome Single Genome Amplification (SGA) approach was employed to generate both the 3′ and 5′ end genomes as previously described [19].

#### 2.2.2. HIV-1 Half Genomes

Briefly, the SGA half genome method entailed amplifying overlapping 5′ (U5, *gag*-*pol* and *vif*) and 3′ (*pol*, *vif*, *vpr*, *rev*, *vpu, tat*, *env*, *nef* and U3-R) half genomes. The generation of cDNA and amplification of HIV-1 half genomes have been previously described [19]. We used primers 5FIV-R1/b5r1 and 1.R3.B3R (Table 2) for 5′ and 3′ half genome to make cDNA, respectively, using superscript IV. Primers used for first-round PCR were RVDA-F1 and 5FIV-R1 for 5′ and b3F1 and 1.R3.B3 (Table 2) for 3′ half genome amplification, respectively. Amplification reactions were performed with 10× High Fidelity Platinum Taq PCR buffer, 10 uM of each primer, 50 mM MgSO_4_, 10 mM of each deoxynucleoside triphosphate and 5 units/µL of Platinum Taq High Fidelity polymerase in reactions of 25 µL (Invitrogen, Carlsbad, CA, USA). The first round of PCR was performed at 94 °C for 2 min, followed by 35 intervals of 94 °C for 15 s, 55 °C for 30 s and 68 °C for 6 min, followed by a final extension at 68 °C for 10 min. The number of cycles was increased to 37 °C for the second round of PCR, and the annealing temperature was adjusted from 55 °C to 58 °C. Primers used for the second round were RVDA-F1 and 5FV-R22 plus b3F3 and 2.R3.B6R (Table 2) for 5′ and 3′ half genomes, respectively.

#### 2.2.3. SMRT Sequencing of HIV-1 Genomes to Identify T/F

Using the Wizard SV Gel and PCR Clean-Up System (Promega, Madison, WI, USA), positive ~9 kb single genome amplicons were gel-extracted. Four SMRTbellTM libraries of near full-length single genome amplicons and 2 SMRTbellTM libraries of half genome amplicons were built to gain deep sequencing data. The PacBio sequencing method was described previously [20]. In brief, we combined 75 near full-length single genome amplicons for each RSII library; 10 patients’ half genome PCR products were collected for each RSII library. The final library DNA concentration was more than 20 ng/µL, purity 260/280 ratio was greater than 1.8 and 260/230 ratio was greater than 2.0; the total volume was 30 µL. SMRT sequencing was performed on PacBio RSII at the University of Delaware DNA Sequencing & Genotyping Center. An algorithm described by Dilernia et al., 2015 [20] that stratifies unique reads from the different genomes and estimates consensus within each genome strata was used to remove sequencing error. To determine the T/F, all ~9 kb viral sequences were aligned using MUSCLE in Geneious bioinformatics software (Biomatters, Auckland, New Zealand), followed by manually aligning. Maximum likelihood parsimony with 100 bootstraps was used for phylogenetic analysis. MEGA7 was used to extract pairwise distances for every intra patient variation using the Poisson correction model. The Los Alamos National Database HIV Consensus/Ancestral Sequence Alignments were used as reference sequences.

#### 2.2.4. Subtype Classification

HIV-1 subtype classification was done using REGA (http://hivdb.stanford.edu/, accessed on 24 December 2021), Recombination Identification Program (RIP) (http://www.hiv.lanl.gov/content/sequence/RIP/RIP.html, accessed on 20–29 March 2021) and jpHMM programs (GOBICS; University of Göttingen) [21,22,23]. The jpHMM tool (http://jphmm.gobics.de/submission_hiv, accessed on 20–29 March 2021) was used to obtain recombination breakpoints, and the recombinant HIV-1 drawing tool from Los Alamos National Laboratories (https://www.hiv.lanl.gov/content/sequence/DRAW_CRF/recom_mapper.html, accessed on 20–29 March 2021) was used to generate the recombinant breakpoint maps. 

### 2.3. Generation of Infectious Molecular Clones and In-Fusion Cloning

**Figure 2:**
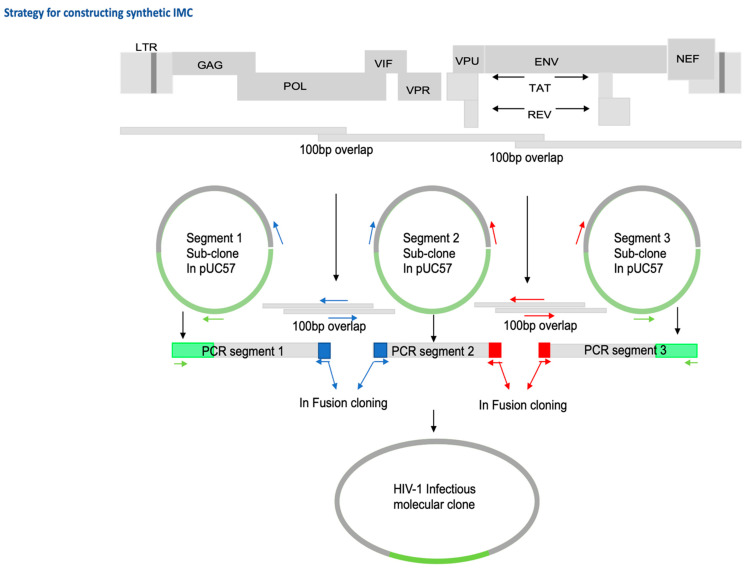
Schematic showing the strategy of constructing synthetic IMC showing 100 bp overlaps in POL and ENV genes.

HIV-1 T/F genomes were chemically synthesized (by GenScript Inc., Piscataway, NJ, USA) in three fragments with 100 bp overlaps in the *pol* and *env* regions (Figure 2) of the proviral genome (to facilitate In-Fusion HD cloning) and ligated separately and, whenever possible, in the same orientation into the multiple cloning site (MCS) of the pUC57 plasmid vector, which contains an ampicillin resistance marker gene for selection. To combine the three genome fragments into one contiguous proviral genome sequence and generate an infectious molecular clone (IMC) plasmid, we utilized an In-Fusion cloning-based approach. The principle of the strategy we followed is outlined below; for some of the IMC, the cloning strategy had to be customized further to overcome plasmid instability challenges that can arise when cloning primary HIV-1 viral proviral genomes. Primers (Table 1) overlapping by 15 to 20 nt in forward (fwd) and reverse (rev) orientation were designed in the ampicillin resistance gene and the overlapping regions in segment 1 and 2 and segment 2 and 3. These were then used to generate 3 PCR fragments using NEB Q5 high-fidelity DNA polymerase: PCR segment 1 contained the portion of pUC57 vector from ampR through proviral segment 1 (~4300 bp); PCR segment 2 spanned proviral segment 2 (~3850 bp), overlapping with the 3′ end of segment 1 and 5′ end of segment 3 by 15–20 nt, respectively; PCR Segment 3 spanned proviral segment 3 and the second portion of pUC57 into the ampR sequence (~4860 bp). PCR fragments were purified using a QiaQuick Gel extraction kit (Qiagen Inc, Valencia, CA, USA) following agarose gel electrophoresis using Invitrogen™ SYBR™ Safe™ DNA Gel Stain and Invitrogen™ Safe Imager™ 2.0 blue-light transilluminator for band visualization. Optimized ratios of purified fragments were subjected to In-Fusion HD Cloning Plus CE (TaKaRa Bio, Mountain View, CA, USA) ligation, principally following the manufacturer’s instructions. 

Two and a half microliters of the In-Fusion cloning reaction above were added to Stellar competent cells, incubated on ice for 30 min and cells were heat shocked for 45 s at 42 °C. Pre-warmed SOC was added to make a final volume of 500 µL and incubated for 1 h at 30 °C while shaking. The transformation reaction was plated on LB plates containing 100 μg/mL of ampicillin or carbenicillin antibiotic, and plates were incubated for 18 h at 30 °C. Individual isolated colonies were picked, sub-cultured at 30 °C overnight and miniprep plasmid purified (PureYield™ Plasmid Miniprep System; Promega, Madison, WI, USA). Multiplex PCR was used to check for the full lengths’ insert of the IMCs. To preempt the deletion of proviral sequences, correctly sized miniprep DNA was usually re-transformed into Invitrogen™ MAX Efficiency™ Stbl2™ competent cells, followed by large-scale culture in carbenicillin-containing LB medium and maxiprep DNA preparation (PureYield™ Plasmid Maxiprep System, Promega). Sequence confirmation of the IMC was done using Sanger sequencing. The 14 TFV sequences that were analyzed as infectious molecular clones (IMCs) are indicated in Table 1 (Genbank accession numbers MW006052 to MW006081).

### 2.4. Generation of Virus Stocks and Determination of Viral Replicative Capacity

Viral stocks of IMCs were generated by transfecting 1.5 µg of purified proviral plasmid DNA into 293 T cells in 6 well plates (American type culture collection) using the Fugene HD transfection reagent (Roche, Basel, Switzerland). Viral stocks were collected 72 h post-transfection, clarified by low-speed centrifugation and frozen at 80 °C. The titer of each viral stock was determined by infecting TZM-bl cells (NIH AIDS Research and Reference Reagent Program) with 5-fold serial dilutions of virus in a manner previously described [24]. In order to assess the in vitro replicative capacity of the IMC, 5 × 105 whole Peripheral blood lymphocytes (PBL) from one donor were infected at a multiplicity of infection of 0.05 (based on TZM-bl titer), and 100 µL of viral supernatants were collected at 2-day intervals during a period of 10 days. Briefly, previously activated peripheral blood leukocytes cells (using DNASE I, Interleukin-2 (IL-2) and phytohemagglutinin) and virus were spinoculated at 37 °C for 2 h, washed 5 times with complete R10 media and a volume of 200 µL was plated into 48 or 96-well U-bottomed plates. Viral supernatants were taken at days 2, 4, 6 and 8 and replaced with an equal volume of complete R10 media containing IL-2. Virion production was quantified using a P33-labeled reverse transcriptase assay as previously described [25]. The optimum window for logarithmic growth for all viruses was estimated to be between days 2 and 6 based on values obtained for days 2–8. By day 8, many high replicating viruses had depleted their target cells, allowing the replication curve to flatten or decrease. As a result, log10-transformed slopes were computed using days 2, 4, 6 and 8 for all viruses. Viral Replication Capacity (VRC) scores were generated by the area under curve normalized with the wild-type subtype C lab adapted strain, MJ4 (AF321523). Each sample was analyzed in triplicate in three independent experiments in order to generate average VRC scores as a measure to control assay variability and avoid a potential bias in determining score values. Included also were the NL4.3 and R880F.

### 2.5. Statistical Analyses

To characterize select T/F IMC, the association between VRC in vitro and patient’s CD4+ T cell decline in vivo, a subset of 14 volunteers with longitudinal CD4+ T cell counts and VRC was studied. The relation between VRC and decrease in CD4+ T cells was estimated using the Mantel–Cox method in Prism software. The time before CD4+ T cell counts that fell below a certain threshold, such as 350 or 500 cells/mm^3^, is known as the endpoint [26]. The difference between set point VL and VRC was also determined using Prism software.

## 3. Results

### 3.1. Study Subjects

A total of 29 adult subjects recruited between 2006 and 2011 in Uganda and whose plasma was collected during the acute/early phase of HIV-1 infection were analyzed in this study. Of the 29 participants (Table 2), 14 (48%) were women and 15 (52%) were men. They represent a section of a young Ugandan heterosexual population with a mean age of 31 (range: 21 to 58) whose major risk factor for HIV infection was co-habitation with a seropositive partner and who became ultimately infected. We examined plasma samples taken near the time of infection, with a mean estimated time from infection (EDI) of 42 days (range: 11 to 73). A significant positive correlation was observed between the TMRCA derived from the 5′ and 3′ end genome data (*p* = 0.006). The mean TMRCA was significantly higher for the 3′ half sequence data relative to the 5′ end (30.65 vs. 52.75, *t*-test *p* = 0.0005).

Viral loads at the first seropositive visit for all 29 samples varied broadly (range: 201 to 1,394,000 copies) with a mean of 238,166 copies/mL. When grouped by gender, women and men did not significantly differ (*p* > 0.05, *t*-test assuming equal variances) in their mean age (29 versus 34 years), EDI (43 versus 41 days) and viral load (294,144 versus 185,919 copies/mL). We next identified single and multivariant transmissions, inferred the nucleotide sequence of the near full-length T/F virus, determined their subtype composition, characterized their sequence variability and determined the VRC using a panel of 14 IMCs that represent the most common subtypes and inter-subtype recombinants transmitted in Uganda during the 2006–2011 time period. 

### 3.2. HIV-1 Genetic Diversity

**Figure 3:**
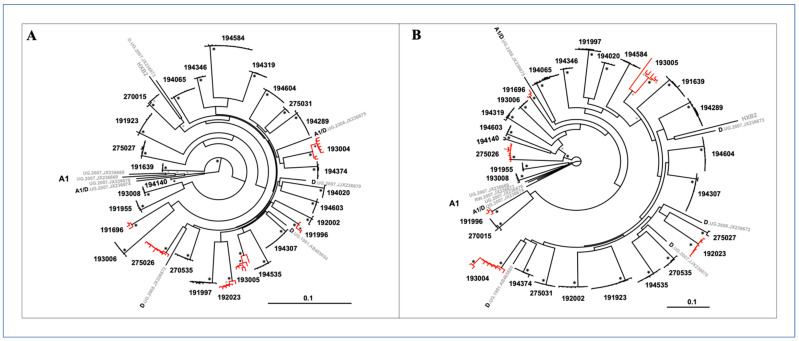
Maximum likelihood phylogenetic trees of half genome sequences from 29 subjects. (**A**) 5′ half genomes. (**B**) 3′ half genomes. Single genome amplification (SGA sequences from each subject fell into distinct monophyletic lineages with low genetic diversity and 100% bootstrap support (black asterisks); nodes with bootstrap support ≥80% shown with grey asterisks. Branches colored in red denote sequences with intra-patient maximum diversity >0.6%; some have sub-lineages with 100% bootstrap support. HIV-1 subtypes A1, D, A/D and subtype B HXB2 reference sequences from the LANL database are shown in grey. The scale bar represents 10% genetic distance.

Viral sequences from 29 study subjects were split into 5′ half and 3′ half genomes for genetic analysis. A total of 209 5′ half genomes (median of seven sequences per subject) and 197 3′ half genomes (median of six sequences per subject) were phylogenetically analyzed. Maximum likelihood phylogenetic trees of 5′ half genomes (Figure 3A) and 3′-half genome nucleotide sequences (Figure 3B) showed distinct monophyletic lineages in a patient-specific pattern with strong statistical support. Sequences from 23 of the 29 subjects formed clusters with very small branches with no or little structure indicative of low intrastrain genetic diversity, strongly suggesting that these infections resulted from transmissions of a single virus or two or more closely related viruses. In contrast, the remaining six other subjects showed sequence clusters with more heterogeneous viral populations, depicted with larger branches and more structure at their tips (shown in red) in both 5′ half and 3′ half phylogenetic trees (Figure 3A,B).

We next employed within-patient sequence diversity and model predictions to identify single and multivariant transmissions. The median number of amplicons per sample was seven (range: 2–14) for 5′ half and six (range: 2–12) for 3′ half genomes (Table 3). Maximum within-patient half genome diversities ranged from 0.07% to 11.01% for 26 individuals; three other subjects were excluded from the diversity analysis because two samples had only two to three sequences in both or at least in one half genome, while the third subject had two out of four sequences highly enriched for APOBEC3G G-to-A mutations. There was a significant correlation between 5′ half and 3′ half maximum diversity values (r = 0.89, *p* < 0.001), and no significant difference between their means values: 5′ half mean 0.38% versus 3′ half mean 0.85% for the 26 participants. Maximum diversity values and conformance to a model of random diversification for single virus transmissions were next examined for 26 individuals as previously reported [12,19,27,28].

### 3.3. Model Predictions Analysis 

**Figure 4:**
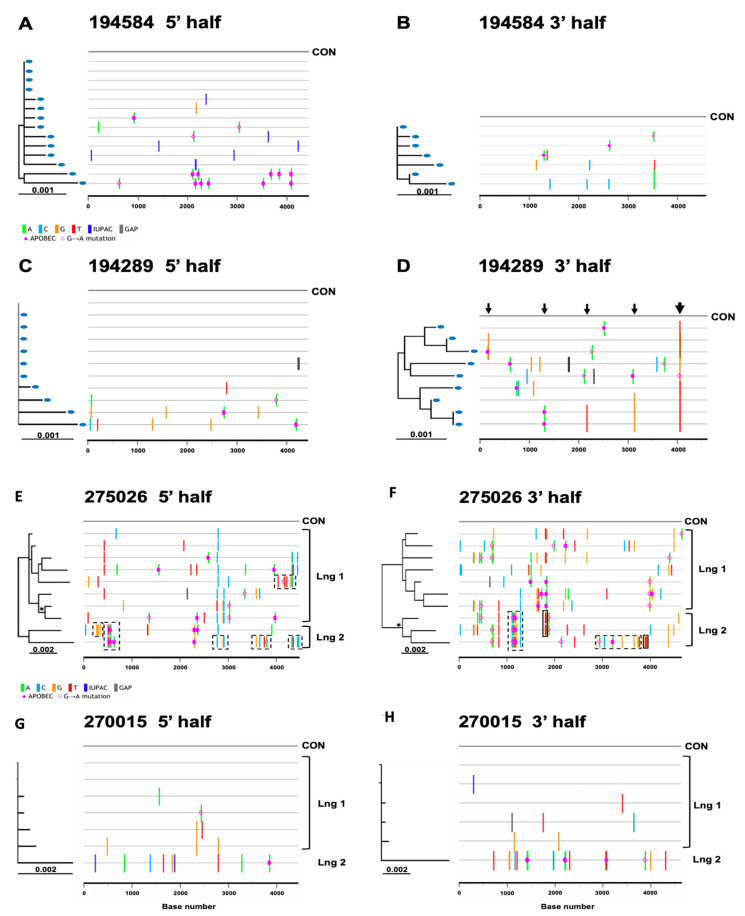
Highlighter plots (**A**–**H**) of two individuals (194584 and 194289, 5′ and 3′ half genomes) with single virus transmission (one unambiguous and one with immune selection), plus two individuals (275026 and 270015, 5′ and 3′ half genomes) with multivariant transmission (one with two sub-lineages and one with a single divergent sequence).

Based on model predictions from previous studies [11,19], the maximum diversity expected within 100 days of transmission of a single virus is 0.6% with confidence intervals of 0.54–0.68%. For all study subjects, clinically determined estimates for the number of days since infection were available, which fell within a range of 11 to 71 days (Table 2), thus allowing us to use previously developed model predictions. Sequence data relevant for predictions are summarized in Table 3. Out of the 26 subjects included in the diversity analysis, 21 had a half genome maximum sequence diversity of <0.6%, whereas five subjects had a sequence diversity greater than 0.6%—the latter being inconsistent with the prediction of an infection initiated by a single virus [11,19]. Phylogenetic and highlighter plot analysis of the 21 subjects displaying <0.6% maximum within-patient sequence diversity allowed us to differentiate those infected by a single virus from those infected by more than one closely related viral variants. We found that sequence data for 19 out of 21 subjects (73%) were indicative of single virus transmission. For example, subject 194584 had sequences with mutations randomly distributed following a Poisson distribution and coalescing to a single consensus in both half genomes (Figure 4A,B); two of the 14 5′ half genomes sequences were hypermutated by APOBEC3G; the removal of G-to-A sites restored the star phylogeny. Another factor causing deviations from a star phylogeny and model predictions of single virus transmission was the appearance of shared polymorphisms in two or more sequences due to early stochastic changes or immune selection. Indeed, subject 194289’s 3′ half genomes (Figure 4D) showed five loci with shared polymorphisms, most notably in the nef gene exhibiting multiple polymorphisms within a region spanning five amino acid residues. This pattern of clustered polymorphisms strongly suggests a vigorous immune response to an epitope in the Nef protein (thicker arrow). Most of the 19 subjects identified as single virus infections had sequences in which confounding factors due to enrichment in APOBEC3G sites and selection were discerned. 

On the other hand, sequence analysis showed that seven out of twenty-six (27%) individuals were most likely to be infected by more than one virus. Two examples of multivariant transmissions are shown next. Subject 275026 displayed half genome sequence diversities that did not conform to model predictions of single virus transmission (Table 3). Phylogenetic and highlighter analyses of 10 viral sequences revealed a non-random distribution of mutations and evidence of two viral sub-lineages distinguished by at least 20–26 polymorphisms in the 5′ and 3′ half genomes (Figure 4E,F). The predominant founder lineage represented by seven sequences and a minor second variant by three sequences are depicted in Figure 4E,F. Polymorphisms identifying the second lineage in subject 275026 were clustered into five segments in 5′ half genomes (see dashed line boxes in Figure 4E), each with 3 to 6 polymorphisms within a region spanning an average length of 159 nucleotides (range: 132–219). Clustered mutations were interspaced by identical or nearly identical sequences to the consensus of the predominant lineage. A similar pattern was seen in the 3′ half genomes. (Figure 4F). We thus inferred that these clustered polymorphisms represent recombination events with a second founder virus still detectable approximately two months after infection. A separate analysis of seven homogeneous 3′ half genome sequences identifying the major lineage (Table 3) conformed to model predictions of single virus transmission. Altogether, the data indicate that subject 275026 was infected by at least two closely related variants from a single donor.

In the case of subject 270015 (Figure 4G,H), the plasma sample was estimated to be taken shortly after infection (11 days), and 5′ and 3′ half genome sequences had low maximum diversities (0.27% and 0.46%, respectively), a random distribution of mutations and a star phylogeny, thus suggesting single virus transmission. However, the Poisson time estimate (>150 days) was not consistent with the clinical data, and sequences did not fit the model prediction of single virus transmission (Table 3). The highlighter plot revealed one sequence containing a high number of polymorphisms (9 in the 5′ half and 19 in the 3′ half) that cannot be explained by APOBEC hypermutation and are not the expected number of mutations within 11 days from infection for a single virus transmission event. We reasoned that the more divergent sequence represents a second founder virus. Indeed, exclusion of this divergent sequence in the analysis showed that the remaining five homogeneous 3′ half sequences (depicted as lineage one in Figure 4H) conformed to model predictions of a single ancestor. Altogether, these data support the idea that subject 270015 was infected by two closely related viruses from the same donor, with lineages diverging from each other by as much as 0.4% in the 3′ half genome. Half genome sequences from five additional individuals also showed evidence of multivariant transmissions, as listed in Table 3 and represented individually in the phylogenetic trees and highlighter plots of half genomes depicted in Supplementary Figure S1. 

Three subjects (191996, 275027 and 194140) had a low number of sequences per sample or exhibited APOBEC-mediated hypermutation and for these reasons were excluded from this analysis. Additionally, these three subjects were also sampled > 50 days from infection.

### 3.4. Inference of Transmitted/Founder Viruses

Next, we inferred the transmitted/founder viral sequences in the 29 study subjects and determined their subtype composition. In the 19 cases of single virus transmissions, star phylogenies and highlighter plots of half genomes coalescing to a consensus sequence allowed us to infer the sequence of the most recent common ancestor for both 5′ and 3′ half genomes. Seven of nineteen subjects (37%) had star phylogenies after excluding or without excluding APOBEC sites (Table 3), thus allowing us to unambiguously infer a T/F virus sequence. As shown in Figure 3A, 5′ half genome sequences (*n* = 14) from subject 194584 were identical or near identical to the consensus with a random distribution of mutations coalescing a consensus except for two sequences with multiple APOBEC3G mutations; removal of the APOBEC mutated sites resulted in a star phylogeny and conformance to model predictions. Analysis of 3′ half genomes (Figure 4B), showed seven near identical sequences, including two sequences with a single shared polymorphism that was identified as an early stochastic change as the model of random evolution predicts to happen in some cases. Thus, the shared mutation did not confound the inference of a consensus sequence in this subject and in other similar cases. Consensus sequences of both 5′ and 3′ half genomes were linked through a partial overlap to complete the near full-length HIV-1 genome. On the other hand, 12 of 19 subjects with single virus transmissions had some sequences harboring few shared polymorphisms that suggested immune selection and confounded the unambiguous inference of the transmitted virus sequence. As there were no pre-sero-conversion plasma samples available from earlier time points, it was not possible to resolve this issue. When shared polymorphism(s) occurred in a fraction of SGA sequences, the predominant nucleotide was assigned in the consensus sequence. This is represented in the 3′ half genome of subject 194289 (Figure 4D) in which two distinct nucleotide sites differed in about half of the nine sequences, and both mutations conferred amino acid changes in the Nef protein. Because these two mutational sites were accompanied with three additional shared polymorphisms in a region spanning five amino acid residues, it strongly suggested immune selection. Four other sites with shared polymorphisms that changed amino acid residues (involving two or three sequences) were detected in other genes: one in *vif*, one in *vpu* and two sites in the env gene; at each site, the predominant nucleotide was assigned in the consensus sequence. On the other hand, when a shared polymorphism occurred in exactly half of the sequences, the ancestral nucleotide was selected. Thus, in 12 study subjects with shared polymorphisms, we could not be 100% certain if the assigned nucleotide truly corresponded to the actual transmitted virus sequence; it is still possible that we inferred an early adapted variant with few mutation changes that emerged under immune pressure or a founder virus with one or two stochastic changes that conferred a replication/survival advantage over the transmitted virus. In any event, in about two-thirds of subjects with single virus infections, we identified an early founder virus that may be one or few nucleotides away from the actual transmitted virus. 

In seven cases of multivariant transmissions, the lineage of one of the transmitted/founder viruses, defined as virus 1 in Figure S1, was identified as the consensus of the set of sequences harboring the least number of mutations. Lineage one was easier to identify in five subjects (275026 and 270015 shown in Figure 4H and 191997, 192023 and 191696 in Figure S1) than in two other individuals whose sequences showed great variability (193004 and 193005 shown in Figure S1). The identification of the second and third transmitted variant was not possible due to the overall low number of sequences exhibiting substantial heterogeneity with multiple adaptation and immune selection mutations as well as recombination events among founder viruses; this was best illustrated for subject 275026 for whom evidence of a second strain is shown in sequences with dashed line boxes in Figure 4E,F. Sequences with a variable number of recombination events between founder viruses were also apparent in subjects 192023, 193004, 193005 and 191696 (Figure S1). Although, the inferred lineage sequence of one founder virus may represent an early virus rather than a transmitted virus.

### 3.5. Subtype Composition

**Figure 5:**
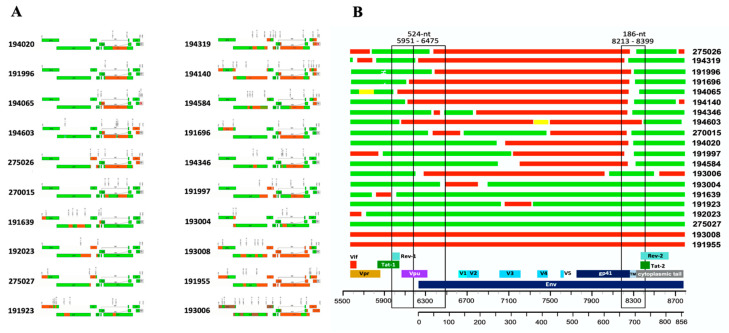
(**A**) Mosaic structures of 20 HIV-1 inter-subtype recombinant T/F viruses. (**B**) Recurrent recombination breakpoints (hotspots) in the envelope region. Subtypes D (green), A1 (red) and C (brown).

Irrespective of whether the inferred consensus represents a bona fide T/F virus or an early virus, the sequence information allowed us to determine the most common subtype composition in our cohort from Uganda. Analysis using different subtyping and recombination analysis tools (see methods) of near full-length genome sequences from 29 T/F viruses showed an overall good agreement between methods. Unique mosaic inter-subtype recombinant strains accounted for most infections (68.9%, 20/29), of which 19 were A1/D and one A1/C/D recombinant as shown in Figure 5A. Infections by pure clade D strains only accounted for about one-third (31%, 9/29) of sero-converters in our cohort. We observed that subtype D tended to predominate in the *gag/pol* gene; in fact, the entire *gag/pol* region and often the *vif* gene were clade D in six out of twenty recombinant viruses, whereas as few as three or four viruses were subtype A1 in approximately half the length of gag/pol genes (Figure 5A). The other pattern observed is that in half of the recombinant viruses, most of the gp120 and part of gp41 were subtype A1, while both exons 1 and 2 of Rev and Tat were almost invariably subtype D. A similar pattern was observed in the envelope region of the single A1/C/D recombinant virus detected, where D and C subtypes were intermixed in the *gag/pol* and *vif* regions. Interestingly, the only two viruses in which the entire envelope gene was subtype A1, exons-1 and -2 of rev and tat genes were also subtype A1.

### 3.6. Recombination Breakpoints

To gain further insights into how A1 and D subtypes recombine, we determined the recombination breakpoints in the 20 inter-subtype recombinant founder viruses inferred above. Unique mosaic inter-subtype recombinant strains accounted for most of the infections (68.9%, 19A1/D and one A1/C/D) as shown in Figure 5A. The complete envelope gene (HXB2 coordinates 6225-8795) covering the ~856 amino acid residues are depicted as mosaic recombinants between clade A1 (shown in red) and clade D (shown in green; Figure 5A). A high rate of recombination was observed within the envelope gene, and an initial analysis suggested that recombination breakpoints were enriched in coding regions for integrase, gp120 (gp160 signal peptide (SP)/vpu overlap and variable V1-V2 loops) and gp41 (Tat2/Rev2 flanking region, encoding the membrane spanning domain) within the 20 unique recombinant TFV analyzed. Two hotspot regions for recombination are suggested by this analysis; the most abundant is within the gp41 transmembrane domain (approximately 75% of the 16 envelopes have this recombination event), and the second is in the signal peptide-C1 region with approximately 25% (4/16) of the samples having this recombination event (Figure 5B). Interestingly, in recombinant proviral genomes, the *gag-pol* coding region is predominantly subtype D, and the directional change between subtypes from 5′ to 3′ end was mostly from subtype D to A to D (Table 4), with this final switch to D occurring within the envelope region. The cytoplasmic tail coding region and the exons 2 of *tat* and *rev* of these recombinant T/F viruses without exception were derived from subtype D. 

### 3.7. Viral Replicative Capacity

**Figure 6:**
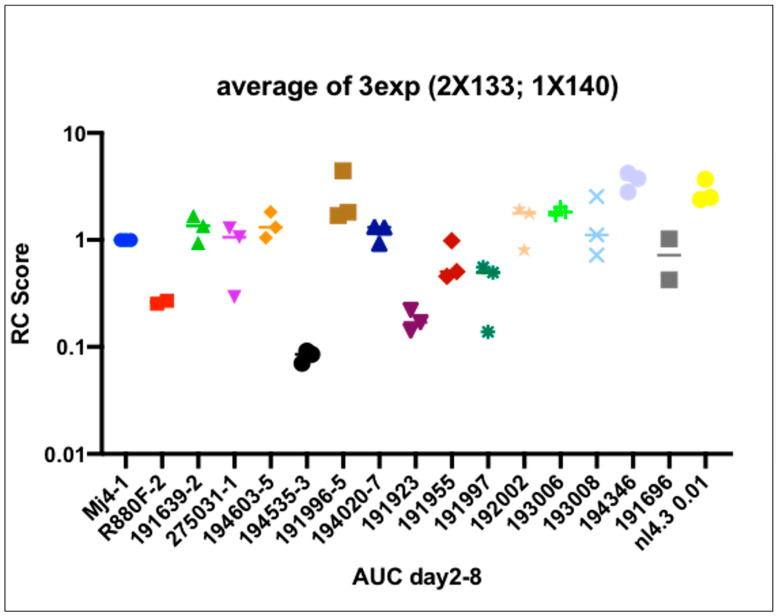
Viral replicative capacity (VRC) scores of 14 infectious molecular clones generated, including the relevant controls, MJ4,NL4.3 and R880F.

VRC scores were determined based on the area under the curve for each sample (Figure 6) and normalized by the MJ4 HIV-1 clone. This clade C chimeric clone (MJ4) was preferred as a control because like the 14 IMC T/F viruses in this study, MJ4 is CCR5-tropic, unlike NL4-3, which is CXCR4-tropic. Additionally, MJ4 has been used in previous studies of VRC and allows comparisons to be made. The lab-recombinant clade B virus, NL4-3, despite being more closely similar to clade D, was not chosen for normalization because its VRC range even at low multiplicity of infection was significantly higher than the primary clade D and A/D viruses in this study. The R880F virus was also included, an example of a poorly replicating HIV-1 IMC. High VRC was defined as the top tercile of T/F virus VRC, while low VRC was defined as the bottom tercile. It is unknown whether viral replicative capability plays a role in HIV-1 transmission. Some research into transmission pairs suggests that T/F viruses have higher replicative fitness than non-transmitted variants, but other studies of T/F variants did not demonstrate increased viral fitness in terms of particle infectivity or viral replicative capacity [13,29]. The replicative capacity of recombinant A/D founder variant infection (*n* = 11) was compared to that of pure D variants (*n* = 3) from these early infections to determine whether there was a difference in replicative capability. The median T/F VRC was 1.211, ranging from 0.085 to 3.75, with 8 T/F VRC scores over the median (Figure S2). The mean VRC scores for A/D and D were 1.307 and 0.975, respectively.

### 3.8. VRC Association with CD4+T Cell Count and Set Point Viral Load

**Figure 7:**
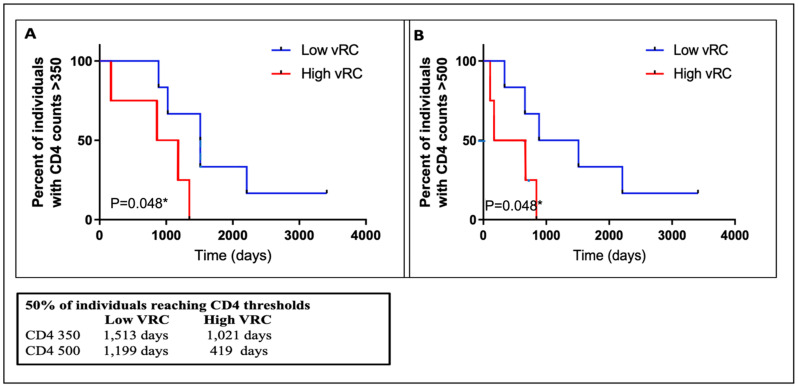
Infection with high replicating viruses resulted in lower CD4+ T cell counts in the first 4–6 years of infection. (**A**) Infection with high VRC resulted in faster drop to CD+T cell counts >350 cells/mm^3^. (**B**) Infection with high VRC resulted in faster drop to CD+ T cell counts >500 cells/mm^3^. * *p* values were calculated using a Mann–Whitney U test for small numbers.

**Figure 8:**
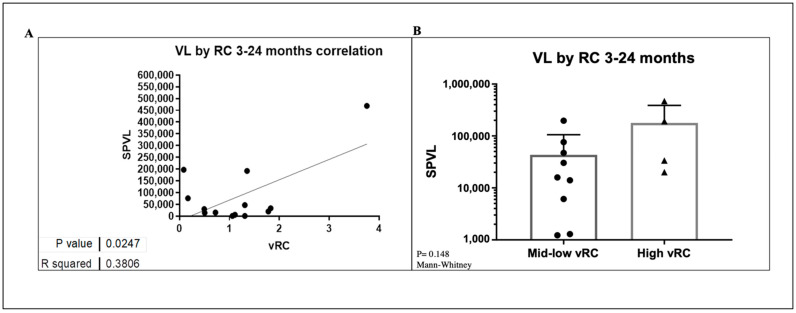
(**A**) A correlation of SPVL versus VRC. (**B**) Differences between low and high VRC and SPVL.

Previous studies have shown that transmitted viral characteristics in subtype C infection significantly correlate with set point viral load (SPVL) as well as CD4 T-cell decline, even in the context of viral control by previously identified host factors [30,31,32]. However, the role of viral replicative capacity in influencing disease progression among subtypes D and A/D recombinant infection has not been adequately studied. Using the Mantel–Cox method in Prism software, Kaplan–Meier curves were compared between two groups of high and low VRC (Figure 7). This analysis showed that the replicative capacity of the initial infecting viral strain had a statistically significant impact on the trajectory of the CD4 counts in the first 4–6 years of follow-up in this small group of ART-naïve participants. The median time to CD4 count <500 cells/mm^3^ was 1199 days for individuals with low VRC and 419 days for those with high VRC. The respective median time to CD4 count <350 cells/mm^3^ was 1021 and 1513 days for individuals with high and low VRC, respectively. In both cases, in individuals infected with HIV strains with high VRC, CD4+ decline was significantly faster compared to those infected with low VRC viruses. Next, we examined the effect of VRC on SPVL. We defined SPVL as the median viral load following acute phase HIV-1 infection (which, in this case, was 3–24 months). A statistically significant positive correlation was observed between the 14 IMC and SPVL (Figure 8A); however, to tease out differences in a small group of individuals, we divided the replicative capacity into high and low VRC as was done previously [26,31]. There was no difference in SPVL between the low and high VRC (Figure 8B). These data are, thus, consistent with previous reports that VRC of the initial infecting strain (T/F) has an impact on some important markers of HIV pathogenesis, especially CD4+ T cell count decline.

### 3.9. Amino Acid Signatures of T/F Sequences 

It has been previously reported [33,34] that His12 in the signal peptide of the Envelope gene is a strong signature for viral transmission. Thus, we examined the amino acid variability at position 12 for the set of T/F genomes described herein. We observed that nearly half (41%; 11/27) of the sequences have the Histidine at position 12 (Table 5). Interestingly, when we compared the peptide signal of different subtypes, subtypes D and A1 were highly contrasting. The subtype D sequences had His12 at a frequency of 70% (14/20), whereas none of the A1 sequences had His12; instead, they had a high frequency (63%; 5/8) for Asn12. None of the subtype D sequences had Asn12. We further observed that His12 was present among recombinants with low VRC (3/5), while recombinants with high VRC (6/9) did not contain His at position 12. 

## 4. Discussion

This study characterized HIV-1 near full-length T/F viral genomes at both the molecular and phenotypic level for mainly subtype D and A/D recombinants from heterosexual mucosal transmissions. HIV-1 mucosal transmission is characterized by an extreme bottleneck that most often results in only one virus variant, termed T/F virus, being selected out of a donor’s quasi-species viruses that cross the mucosal barrier and establish clinical infection in the new host [11,19]. These breakthrough viruses may have unique properties that confer a higher probability to be transmitted [14,17]. Here, we report single viral variant transmissions in 73% (19/26) of the new infection and multiple transmissions in 27% (7/26) of the cases. The proportion of multivariant transmission in our study, although slightly higher, was not statistically different when compared to previous studies in heterosexual populations, which reported proportions ranging from 17.7% to 21.7% [11,12,35]. One of the caveats in our study with regard to assessment of the multiplicity of infection was the limited number of sequences we were able to obtain for some samples that did not conform to the criteria of single variant transmission. Additionally, a later sampling time is expected to impact the detection of transmitted variants that are less fit than a co-transmitted variant that becomes the predominant strain [36]. Thus, it is more likely that an infrequent outcompeted variant would be missed with a limited number of sequences. The power analysis predicted that for samples with only six sequences, the probabilities of multivariant detection were 60% for variants with ≥15% frequency and 80% probability for variants with ≥20% frequency. Nonetheless, the genetic analyses of both 5′ and 3′ half genomes were concordant in all seven individuals with multivariant transmission, cross-validating each other’s findings despite the relatively low number of sequences per sample. Our study did not show evidence of an association between multivariant transmission and inflammation caused by sexually transmitted infections. This finding was unlike previous studies by Halaand and colleagues published in 2009, where multivariant transmissions for subtypes A and C were correlated with genital inflammation.

However, for the majority of study subjects classified as single virus transmissions, we had obtained at least six half-genome sequences (either both or one of the two halves). It is reasonable, therefore, to assume that some, if not all of these samples are single virus infections for vaccine-design purposes because these are the fittest strains that a vaccine should target for neutralization. However, we cannot rule out the possibility that a few individuals may have been infected by a second variant whose less fit progeny represent <10% of the virus population. 

In this study, of 29 subjects initially classified as subtype D using a small fragment of the HIV-1 pol gene, unique mosaic inter-subtype recombinant strains accounted for most infections (69.0%, A1/D or A1/C/D) using near full-length sequences. This proportion reported in our study is higher than previous studies centered on near full-length HIV-1 sequences in Uganda from chronically infected individuals, for which the reported prevalence of HIV-1 inter-subtype recombinants was 46% (92/200) [9], 39.3% (108/275) [37], 30% (14/46) [8] and recently, 49.9% (232/465) [10]. The difference in percentages of recombinants could be due to the limited cohort size in our study (*n* = 29), which focused mainly on the unique sequences corresponding to T/F viruses. Overall, our study, together with others, suggest a high frequency of inter-subtype recombinants in Uganda, most of which are URFs combining subtypes A and D. The extraordinary viral diversity and highly pathogenic nature seen among some of these T/F URFs in our study poses a challenge for both vaccine development and treatment and supports that vaccine products must be matched to the predominant subtype in a country instead of universal vaccines. The virus that crossed the mucosal barrier to establish clinical infection in the new host were, indeed, mainly unique recombinant forms of A1D, suggesting that these recombinant variants must exhibit a selective advantage for transmission over the parental subtypes, but the molecular basis for this property is not yet fully understood.

Interestingly, two hotspots for recombination were identified in the gp41 transmembrane coding region and signal peptide-C1 regions, respectively, as has been previously observed for different subtypes [38,39]. Furthermore, the directionality (5′ to 3′ of the NFL recombinant genomes) of the subtype switches was from D to A1 to D, with the final switch back to D occurring within the envelope transmembrane region. This recurrence of recombination patterns would suggest that generation of a fit hybrid A1D virus may not be a non-random process, but that there must be structural and functional constraints that select for a virus with transmission fitness. In addition, the selection observed for the rev and tat genes, that results in exons 1 and 2 of *tat* and *rev*, respectively, belonging to the same subtype (D in this case) but interspaced by a region of subtype A1, seems not to be a random event, but rather one explainable by biological constraints. The second exons of *tat* and *rev* overlap with the gp41 coding area of HIV-1 *env* and must be appropriately spliced to create functional Tat and Rev proteins, which are two key viral regulatory factors for HIV gene expression [40,41]. Inter-subtype discordance between *tat* and *rev* 1 and 2, respectively, might provide a functional bottleneck for Envelope recombinants with breakpoints upstream of *tat* 2/*rev* 2 exons. Constraints in the overlapping *env* open reading frame could favor this selection of the same subtype in exons 1 and 2 of *tat* and *rev*, but also, this occurrence could be due to pressure on the *tat* [42,43]; in chimeras where 5′ is clade D, it may be advantageous if that is also D. Furthermore, subtype recombinants that have an Envelope cytoplasmic tail that matches the subtype of the *gag* may be advantageous for virion incorporation of Envelope [44]. In addition, we observed that a previously reported amino acid signature, a histidine at position 12 (His12) in the Envelope signal peptide, was highly prevalent in subtype D Envelopes in our study, while the subtype A1 Envelope signal peptide completely lacked His12, and asparagine was predominant at this position. Presence of Histidine at position 12 occurred in virus strains with low viral replicative capacity of the virus, while departure from the Histidine in position 12 was seen for strains with high viral replicative capacity in this study. More detailed phylogenetic analyses will need to be conducted before conclusions on possible associations can be drawn. It is, however, of note that the transmitted Envelope glycoproteins from HIV-1 subtype B with a basic amino acid position 12 were found to be incorporated into virions at a higher density and had higher infectious titers than non-His12 signature envelopes, according to Asmal et al. 2011 [33]. Similarly, the Envelope His12 signature was identified by Gnanakaran et al. 2011 [34], and its expression levels were implicated in selection at viral transmission or early expansion. Thus, further exploration of this important signature with more sequences from acute infection among subtypes A, D and A/D recombinants is warranted.

HIV-1 transmission favors viruses with high infectivity and replication capacity, and a subject’s founder virus replication capacity can predict the rate at which subtype C disease progresses [13,26,31,45]. While there was no significant difference in replicative capacity between subtype D and A/D in this study, replicative capacity of the initial infecting viral strain had a statistically significant impact on the trajectory of the CD4 counts in the first 4–6 years of follow-up in this small group of ART-naïve participants. On the contrary, there was no difference between high and low replicative viruses and SPVL in this study, although there was a significant correlation between VRC and SPVL. Additional analyses with more numbers and representation for subtypes A, D and ADs will be more informative.

## 5. Conclusions

For subtype determination, subtyping based on single partial genome region is inaccurate, as many of the recombinants identified were previously missed when only sequencing *pol*. Therefore, there is a need to use a full genome sequence, or at least multiple regions, for accurate subtyping of the viruses. The HIV-1 Envelope recombination patterns observed in this study further underpin the need for larger studies of HIV-1 acutely infected individuals, given that subtype-specific immunogens are being considered for vaccine development. The numbers of T/F sequences available has significantly increased over time, with a fair representation from all subtypes enabling the extensive bioinformatics analyses with chronic viral sequences to allow for the identification of functional sequence domains unique to T/F viruses. Additionally, the full-length genome infectious molecular clones derived here will be further utilized in mucosal studies to elucidate mucosal transmission biology and to inform other immuno-pathogenesis studies currently ongoing within the IAVI consortium.

## Figures and Tables

**Table 1:** Uganda protocol C cohort and HIV-1 characteristics.

SN	SAMPLE-ID	Sample Date	EDI *	Days Post Infection *****	Initial Viral Load(Copies/mL)	IMC-VRC Score **	Gender	Age ***	Subtype (Full Genome)	GenBank No.
1	194065	12-Jan-10	01-Dec-09	42	254,000	N/A ****	M	41	A/D	MW006066
2	194289	29-Mar-10	14-Feb-10	43	1,394,000	N/A	F	24	D	MW006068
3	193006	07-Apr-09	15-Feb-08	51	112,000	1.84	M	24	A/D	MW006062
4	194604	28-Mar-11	12-Feb-11	44	254,516	N/A	F	35	D	MW006076
5	194603	21-Jul-10	30-May-10	52	177,000	1.38	F	33	A/D	MW006075
6	191696	11-Sep-07	23-Jul-07	50	435,000	0.72	M	29	A/D	MW006053
7	194535	16-Jun-11	30-Apr-11	47	289,930	0.08	M	39	D	MW006073
8	275026	10-Sep-08	21-Jul-08	51	412,000	N/A	F	21	A/D	MW006079
9	194319	09-Mar-09	18-Feb-09	19	602,000	N/A	F	26	A/D	MW006070
10	192023	28-Jan-08	06-Dec-07	53	996,000	N/A	M	27	A/D	MW006059
11	191923	24-Jan-08	30-Nov-07	55	198,000	0.17	F	31	A/D	MW006054
12	193004	30-Oct-07	26-Sep-07	34	6690	N/A	F	25	A/D	MW006060
13	193005	06-Nov-07	11-Oct-07	26	90,500	N/A	M	22	D	MW006061
14	194020	12-Jun-09	07-May-09	36	77,700	1.16	M	33	A/D	MW006064
15	194346	31-Mar-09	28-Feb-09	31	259,000	3.58	M	29	A/D	MW006071
16	191639	03-Apr-08	13-Feb-08	50	102,000	1.32	M	50	A/D	MW006052
17	270015	19-Dec-06	08-Dec-06	11	19,300	N/A	M	58	A/D	MW006077
18	193008	15-Jun-09	23-May-09	23	50,500	1.46	M	27	A/D	MW006063
19	191955	26-Mar-07	03-Mar-07	23	4190	0.64	F	39	A/D	MW006055
20	191997	15-Jan-08	19-Nov-07	57	4610	0.37	M	31	A/D	MW006057
21	192002	24-Jul-07	04-Jun-07	50	2420	1.54	F	27	D	MW006058
22	275031	05-Jun-09	11-May-09	25	3750	0.87	M	31	D	MW006081
23	191996	19-Sep-08	26-Jul-08	55	201	2.64	F	37	A/D	MW006056
24	194307	2-Mar-09	3-Feb-09	27	930,000	N/A	F	21	A/D	MW006068
25	194374	04-Feb-10	24-Dec-09	42	43,300	N/A	M	33	D	MW006072
26	194584	05-Jul-10	03-Jun-10	32	76,700	N/A	F	33	A/D	MW006079
27	270535	17-Dec-08	05-Oct-08	73	51,200	N/A	M	31	D	MW006078
28	194140	28-May-09	07-Apr-09	51	45,600	N/A	F	26	A/D	MW006067
29	275027	01-Oct-08	01-Aug-08	61	14,700	N/A	F	22	A/D	MW006080

* Estimated date of infection ** VRC measured by P^33^-labeled reverse transcriptase assay (see Methods) *** Age at time of sample collection **** N/A: not available ***** No. of days between EDI and the sample date.

**Table 2:** Primers used for cDNA synthesis, amplification and infectious molecular clone (IMC) generation.

Primer Name	Primer Sequence	Primer Use
UG1.3′3_PlCb	5′-ACTACTTAAAGCACTCAAGGCAAGCTTTATTG-3′	cDNA synthesis for near full-length
UGOFM19	5′-GCACTCAAGGCAAGCTTTATTGAGGCTTA-3′	cDNA synthesis for near full-length
1U5Cc	5′-CCTTGAGTGCTTTAAGTAGTGTGTGCCCGTCTGT-3′	Near full-length genome PCR
UG1.3′3_PlCb	5′-ACTACTTAAAGCACTCAAGGCAAGCTTTATTG-3′	Near full-length genome PCR
2U5Cd	5′-AGTAGTGTGTGCCCGTCTGTTGTGTGACTC-3′	Near full-length genome PCR
UG2.3′3′plCb	5′-TAAAGCACTCAAGGCAAGCTTTATTGAGGCTTA-3′	Near full-length genome PCR
5FIV-R1	5′-CTYTTTCTCCTGTATGCAGACCCC-3′	cDNA synthesis for 5′ half genome
b5r1	5′-CTTGCCACACAATCATCACCTGCCAT-3′	cDNA synthesis for 5′ half genome
1.R3.B3R	5′-ACTACTTGAAGCACTCAAGGCAAGCTTTAT-3′	cDNA synthesis for 3′ half genome
RVDA-F1	5′-GGGTCTCTCDGTTAGACCAGAT-3′	5′ half genome PCR
b3F1	5′-ACAGCAGTACAAATGGCAGTATT-3′	3′ half genome PCR
5FV-R22	5′-CCTAGTGGGATGTGTACTTCTGAAC-3′	5′ half genome PCR
b3f3	5′-TGGAAAGGTGAAGGGGCAGTAGTAATAC-3′	3′ half genome PCR
2.R3.B6R	5′-TGAAGCACTCAAGGCAAGCTTTATTGAGGC-3′	3′ half genome PCR
**Example of strain-optimized primers**		
con1.rev	5′-TCTGATGCTTTTTGTCTGGTGT-3′	Generation of IMC (overlap of Fragments 1 & 2)
con1.fwd	5′-ACACCAGACAAAAAGCATCAGA-3′	Generation of IMC (overlap of Fragments 1 & 2)
318.con2.fwd	5′-CCATGTGTAAAGCTAACCCCACTC-3′	Generation of IMC (overlap of Fragments 2 & 3)
318.con2.rev	5′-GGTTAGCTTTACACATGGTTTTAGAC-3′	Generation of IMC overlap of Fragments 2 & 3)
AmpR.PvuI.fwd	5′-CGATCGTTGTCAGAAGTAAGTTGGCCGCAGTGTT-3′	Generation of IMC (amp^R^)
AmpR.PvuI.rev	5′-TTCTGACAACGATCGGAGGACCGAAGGAGCTAACCGCTT-3′	Generation of IMC (amp^R^)

**Table 3:** Diversity analysis of HIV-1 half genomes derived from 29 recent sero-converters.

Subject ID	HIV Half Genomes (a)	Total Number HIV Genomes	Minimum nt Length of Viral Genome	Maximum nt Length of Viral Genome	Nucleotide Sequence Diversity (mean %)	Nucleotide Sequence Diversity (Range %)	Number of Genomes Analysed (b)	Mean HD	Max HD	Poisson Estimated Days Since MRCA (c)	Lambda (d)	Goodness of Fit *p*-Value (e)	HD Fit to Poisson	Star Phylogeny	Deviation from Star Phylogeny	Number of Transmitted Viruses
275026	5′	10	4474	4474	0.41	0.41–0.82	10	16	29	153 (103–202)	16.27	2.00 × 10^−16^	No	No	Multiple strains	≥2
	3′	10	4595	4628	0.57	0.24–0.97	10	24	41	217 (175–258)	23.98	2.00 × 10^−16^	No	No	Multiple strains	≥2
	3′ lng1	7	4595	4616	0.41	0.24–0.55	7	18	25	160 (131, 189)	17.62	0.8246	Yes	No	Selection	Lng 1
270015	5′	7	4449	4449	0.10	0–0.27	7	4.6	12	43 (6, 81)	4.571	1.80 × 10^−5^	No	Yes		≥2
	3′	6	4622	4623	0.16	0–0.46	6	8	21	73 (22, 167)	8	2.00 × 10^−16^	No	Yes		≥2
	3′ lng1	5	4622	4623	0.03	0–0.07	5	2	3	18 (8, 29)	2	0.6198	Yes	Yes		Lng 1
192023	5′	6	4448	4448	0.48	0.16–0.99	6	19	34	184 (80, 288)	19.47	2.00 × 10^−16^	No	No	Multiple strains	≥2
	3′	6	4604	4607	0.54	0.17–0.72	6	23	32	206 (154, 258)	22.6	2.00 × 10^−16^	No	No	Multiple strains	≥2
193004	5′	8	4460	4494	0.87	0.36–1.41	8	36	60	340 (242, 439)	36.46	2.00 × 10^−16^	No	No	Multiple strains	≥2
	3′	8	4636	4673	1.67	0.51–3.39	8	Too high					No	No		≥2
193005	5′	6	4482	4485	1.22	0.31–2.30	6	49	87	456 (187, 724)	48.67	2.00 × 10^−16^	No	No	Multiple strains	≥2
	3′	6	4469	4678		0.20–11.01	6	Too high					No	No	Multiple strains	≥2
191696	5′	3	4450	4450	0.64	0.34–0.89	3	N/A					No	No	Multiple strains	≥2
	3′	3	4575	4602	0.54	0.13–0.77	3	N/A					No	No	Multiple strains	≥2
191997	5′	9	4466	4468	0.20	0.02–0.33	9	5.8	13	62 (42, 83)	5.778	0.00941	No	No (f)	Selection	≥2
	3′	10	4651	4664	0.25	0–0.41	10	11	18	97 (75, 119)	10.78	2.00 × 10^−16^	No	No	Selection	≥2
194037	5′	12	4456	4456	0.04	0–0.11	12	1.7	5	16 (9, 23)	1.667	0.9321	Yes	Yes		1
	3′	12	4596	4599	0.06	0–0.15	12	2.6	7	24 (14, 34)	2.636	0.9332	Yes	Yes		1
194584	5′	14	4455	4456	0.08	0–0.20	14	1.6	4	17 (11, 23)	1.571	0.7648	Yes	Yes (f)		1
	3′	7	4580	4580	0.07	0.02–0.15	7	3.3	7	31 (15, 46)	3.333	0.9464	Yes	Yes		1
270535	5′	8	4459	4460	0.11	0.04–0.18	8	4.9	8	47 (34, 59)	4.929	0.6833	Yes	Yes		1
	3′	5	4576	4601	0.11	0.09–0.13	5	5.2	6	48 (40,56)	5.2	0.3723	Yes	Yes		1
191639	5′	6	4458	4459	0.04	0–0.09	6	2	4	19 (7, 31)	2	0.7987	Yes	Yes		1
	3′	8	4618	4619	0.19	0.07–0.43	8	3.6	8	38 (20, 56)	3.607	0.8119	Yes	yes (f)		1
194603	5′	6	4461	4461	0.10	0–0.22	6	3	7	33 (5, 60)	3	0.8227	Yes	Yes (f)		1
	3′	6	4623	4626	0.19	0.11–0.22	6	5.3	7	55 (40, 70)	5.333	0.8391	Yes	yes (f)		1
275031	5′	4	4547	4465	0.04	0.02–0.07	4	2	3	19 (4, 33)	2	0.6822	Yes	Yes		1
	3′	6	4605	4606	0.07	0.02–0.13	6	2.3	4	24 (13, 35)	2.333	0.5462	Yes	yes (f)		1
194604	5′	10	4459	4459	0.03	0–0.09	10	1.2	4	11 (4, 19)	1.2	0.5375	Yes	Yes		1
	3′	10	4575	4575	0.07	0.02–0.18	10	3.3	8	30 (17, 40)	3.289	0.7812	Yes	No	Selection	1
192002	5′	4	4191	4477	0.16	0.07–0.26	4	7	11	66 (22, 110)	7	0.9283	Yes	Yes		1
	3′	8	4549	4595	0.17	0.02–0.33	8	8.2	15	75 (48, 102)	8.214	0.898	Yes	No	Selection	1
194535	5′	7	4459	4459	0.08	0.02–0.11	7	3.4	5	32 (21, 44)	3.429	0.6776	Yes	Yes		1
	3′	7	4624	4818	0.17	0.11–0.30	6	5.8	8	51 (43, 58)	5.8	0.6561	Yes	No (f)	Selection	1
194319	5′	7	4461	4461	0.05	0–0.09	7	2.3	4	22 (17, 26)	2.286	0.7703	Yes	No	Early selection	1
	3′	7	4575	4593	0.10	0.07–0.13	7	4.4	6	40 (32, 48)	4.381	0.4586	Yes	No	Early selection	1
193006	5′	6	4452	4452	0.07	0.02–0.11	6	3	5	28 (15, 42)	3.0	0.8046	Yes	Yes		1
	3′	6	4435	4489	0.11	0.04–0.18	6	5.2	8	49 (29, 68)	5.2	0.4767	Yes	No	Early stochastic	1
194020	5′	5	4463	4463	0.06	0–0.13	5	2.8	6	27 (-3, 56)	2.8	0.6693	Yes	Yes		1
	3′	5	4623	4623	0.19	0.14–0.22	5	8.5	10	77 (68, 87)	8.5	0.4971	Yes	No	Selection	1
194346	5′	5	4460	4460	0.06	0–0.11	5	2.8	5	27 (9, 44)	2.8	0.7886	Yes	Yes		1
	3′	5	4599	4602	0.14	0.11–0.20	5	6.8	10	62 (44, 80)	6.8	0.8729	Yes	No	Selection	1
194374	5′	7	4455	4458	0.09	0–0.16	7	3.8	7	36 (24, 49)	3.81	0.7509	Yes	No	Early selection	1
	3′	6	4638	4638	0.07	0–0.17	6	3.3	8	30 (10, 50)	3.267	0.657	Yes	No	Early selection	1
191955	5′	6	4473	4475	0.10	0.07–0.13	6	4.7	6	44 (33, 55)	4.667	0.4987	Yes	Yes		1
	3′	6	4630	4642	0.11	0–0.26	6	5	12	45 (6, 85)	5	0.00015	No	Yes		1
191923	5′	11	4461	4461	0.11	0–0.36	11	1.8	6	20 (9, 31)	1.818	0.3695	Yes	Yes		1
	3′	11	4490	4613	0.12	0–0.24	11	5.2	11	47 (30, 64)	5.164	2.08 × 10^−13^	No	No	Selection	1
194065	5′	11	4459	4459	0.06	0–0.16	11	2.9	7	28 (14, 41)	2.909	0.4872	Yes	Yes		1
	3′	11	4591	4594	0.22	0–0.33	11	9.2	15	84 (68, 101)	9.2	0.00063	No	No	Selection	1
194289	5′	11	4425	4446	0.05	0–0.20	11	2.2	9	21 (6, 35)	2.182	1.23 × 10^−8^	No	Yes	yes	1
	3′	9	4599	4614	0.18	0–0.26	9	7.6	11	70 (57, 82)	7.639	0.00011	No	No	Selection	1
193008	5′	5	4491	4492	0.09	0.04–0.16	5	4.2	7	39 (21, 58)	4.2	0.9419	Yes	No	Early stochastic	1
	3′	6	4667	4670	0.18	0.02–0.29	6	8.7	13	78 (53, 103)	8.667	0.00063	No	No	Selection	1
275027	5′	9	4455	4455	0.15	0–0.29	9	6.4	12	60 (42, 79)	6.389	2.00 × 10^−16^	No	No	Selection	1
	3′	2	4302	4641	0.26	N/A	2	N/A								?
191996	5′	4	4459	4461	0.51	0.04–0.75 *	4	N/A. Two out of four sequences were hypermutated by APOBEC3G (A3G)						N/A	A3G Hypermutation	?
	3′	3	4633	4634	0.13	0.11–0.15	3	N/A						Yes		
194140	5′	2	4457	4457	0	N/A	2	N/A								?
	3′	2	4677	4677	0.02		2									

Four samples listed at the bottom of the table were excluded from this analysis because of the extremely low number of 3′ half genome sequences. (a) Genomes (5′ half or 3′ half) from all amplicons or selected sequences identifying the most predominant transmitted lineage (lng1) were analyzed separately. (b) Number of genomes analyzed for conformance to a Poisson model of random diversification. The Poisson-Fitness v2 tool (https://www.hiv.lanl.gov/content/sequence/POISSON_FITTER/pfitter.html, accessed on 20–29 March 2021) automatically screens for enrichment of APOBEC G-to-A substitutions. APOBEC positions were removed when appropriate. (c) Poisson model prediction of the minimum number of days needed to achieve the observed intra-strain diversity. (d) Lambda is the parameter of the Poisson distribution that best fits the HD frequency counts. Lambda value should be close to the mean HD. (e) Low goodness-of-fit (GOF) *p* values (<0.05) indicate divergence from a Poisson; when the maximum Hamming Distance is too high, the Poisson-Fitter may fail. Conversely, a high *p* value indicates conformance to a Poisson distribution. (f) APOBEC sites were removed.

**Table 4:** Inter-subtype recombination events and identification of recombination hot spots in envelope genes of HIV-1 transmitted/founder variants from Uganda.

Unique Identifier	No. of Break Points	Breakpoint Interval	Length, No. Nucleotides	Directionality of Recombination	Envelope Region of Recombination Events	Recombination Hotspot #	vpu	Subtype Composition of Overlapping vpu, tat and Rev Genes/Exons	
		Start–End, HXB2 Numbering						tat-1, rev-1	tat-2, rev-2
194319-env	1	8214–8252	38	A1/D	**TM domain, gp41**	#1	A1/D	D	D
194140-env	1	8251–8313	62	A1/D	**TM domain, gp41**	#1	A1	D	D
194065-env	1	8258–8358	100	A1/D	**TM domain, gp41**	#1	A1	D	D
191696-env	1	8251–8303	52	A1/D	**TM domain, gp41**	#1	A1/D	D	D
194584-env	2	6990–7202	212	D/A1	V3, CD4/co-receptor binding		D	D	D
		8252–8330	78	A1/D	**TM domain, gp41**	#1			
194020-env	2	6975–7057	82	D/A1	CD4 contact, near V3		D	D	D
		8252–8301	49	A1/D	**TM domain, gp41**	#1			
191997-env	2	7116–7135	19	D/A1	V3 loop		D	D	D
		8213–8305	92	A1/D	**TM domain, gp41**	#1			
191996-env	2	6336–6364	28	D/A1	Signal peptide/C1	#2	D	D	D
		8272–8299	27	A1/D	**TM domain, gp41**	#1			
275026-env	3	6326–6360	34	D/A1	Signal peptide/C1	#2	D	D	D
		8270–8333	63	A1/D	**TM domain, gp41**	#1			
		8722–8740	18	D/A1	LLP-1				
194603-env	3	7329–?	?	A1/D	CD4 binding		A1	D	D
		7473–?	?	D/A1	Coreceptor binding				
		8386–8399	13	A1/D	**TM domain, gp41**	#1			
270015-env	4	6309–6351	42	D/A1	Signal peptide/C1	#2	D	D	D
		6622–6674	52	A1/D	V1 loop				
		7470–7506	36	D/A1	CD4/coreceptor binding				
		8251–8297	46	A1/D	**TM domain, gp41**	#1			
194346-env	4	6339–6365	26	D/A1	Signal peptide/C1	#2	D	D	D
		6427–6468	41	A1/D	Signal peptide/C1	#2			
		6745–6779	34	D/A1	V2				
		8246–8296	50	A1/D	**TM domain, gp41**	#1			
193006-env	1	8031–8074	43	A1/D	gp41 fusion domain		A1/D	D	D
193005-env	2	7313–7327	14	D/A1	CD4 binding loop		D	D	D
		7519–7540	21	A1/ D	Coreceptor binding				
193004-env	2	6426–6475	49	D/A1	Signal peptide/C1	#2	D	D	D
		6795–6889	94	A1/ D	V2 loop				
191923-env	2	7018–7055	37	D/A1	CD4 contact, near V3		D	D	D

**Table 5:** Histidine signatures across the various clades within the Envelope gene.

*Env* Subtype	Amino Acid (Single Nucleotide Code)	Sample ID
D	H	194604
D	H	194535
D	H	192023
D	H	191923
D	H	194020
D	H	270015
D	H	191997
D	H	275031
D	H	191996
D	H	194307
D	H	194374
D	N/A	193004
D	N/A	193005
D	Q	192002
D	Q	275027
D	G	194289
D	I	194584
D	L	270535
D	T/P	275026
D	Del	191639
D	D	191955
A1	N	193006
A1	N	194603
A1	N	191696
A1	N	194319
A1	N	194140
A1	D	194346
A1	D	194065
A1	Y	193008

## Data Availability

The sequence data is available in GenBank (See Table 1).

## Supplementary Materials

The following supporting information can be downloaded at: https://www.mdpi.com/article/10.3390/v14020334/s1, Figure S1: Highlighter plots for the five additional samples with multivariant transmissions: (A) 5′ half genomes; (B) 3′ half genomes; Figure S2 Viral replicative capacity scores among the A/D versus the D subtypes. A two tailed Mann–Whitney test was used to test if medians between the two groups were different.
