# Supplementary figures and images for: Characterization of Near Full-Length Transmitted/Founder HIV-1 Subtype D and A/D Recombinant Genomes in a Heterosexual Ugandan Population (2006–2011)
Source: Viruses. 2022 Feb 7;14(2):334. doi: 10.3390/v14020334 (PMC8874453; doi:10.3390/v14020334)

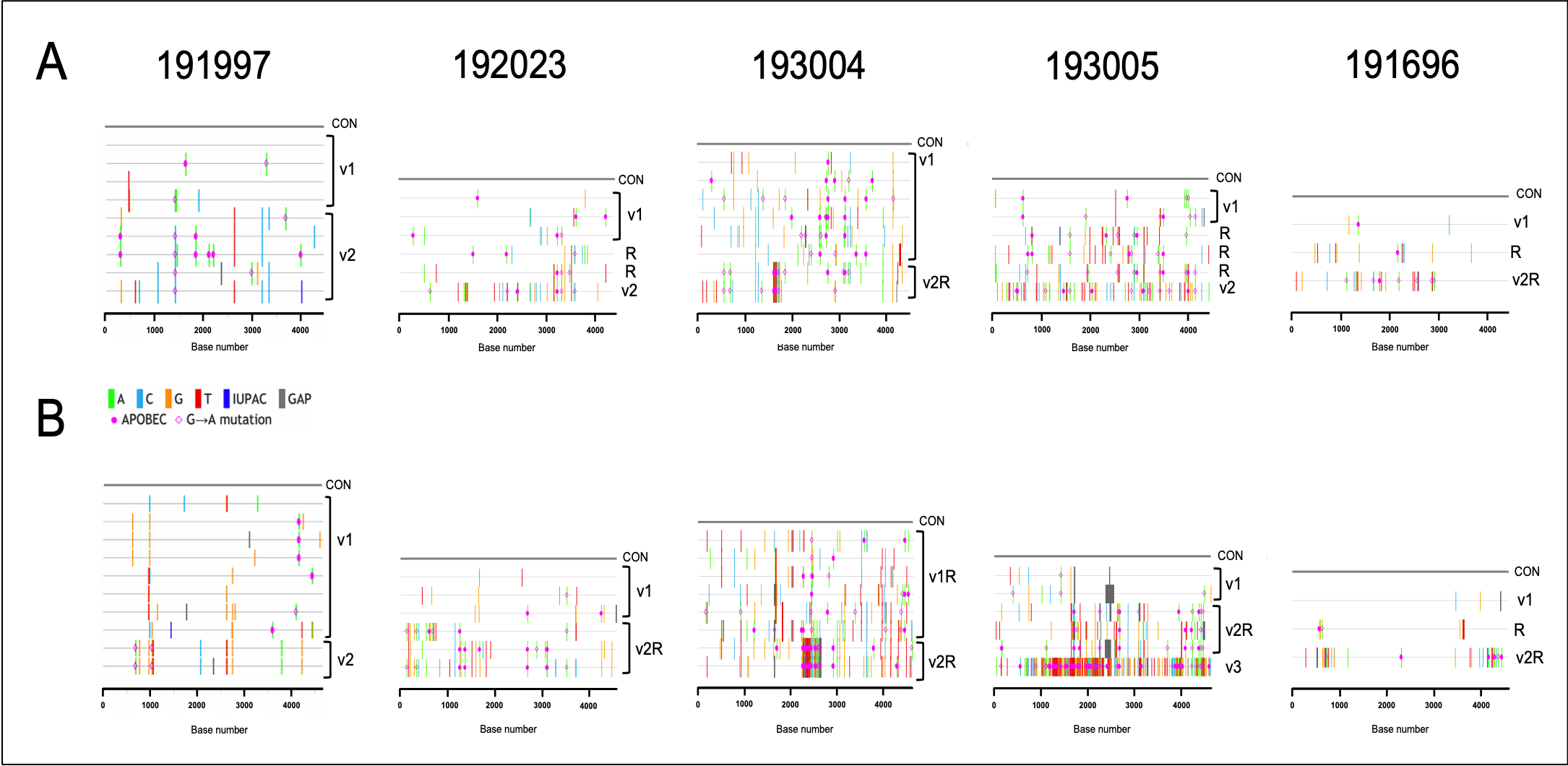

Supplement: Supplementary file 1 [file viruses-14-00334-s001.zip › viruses-1501758-supplementary/S 1.tif]

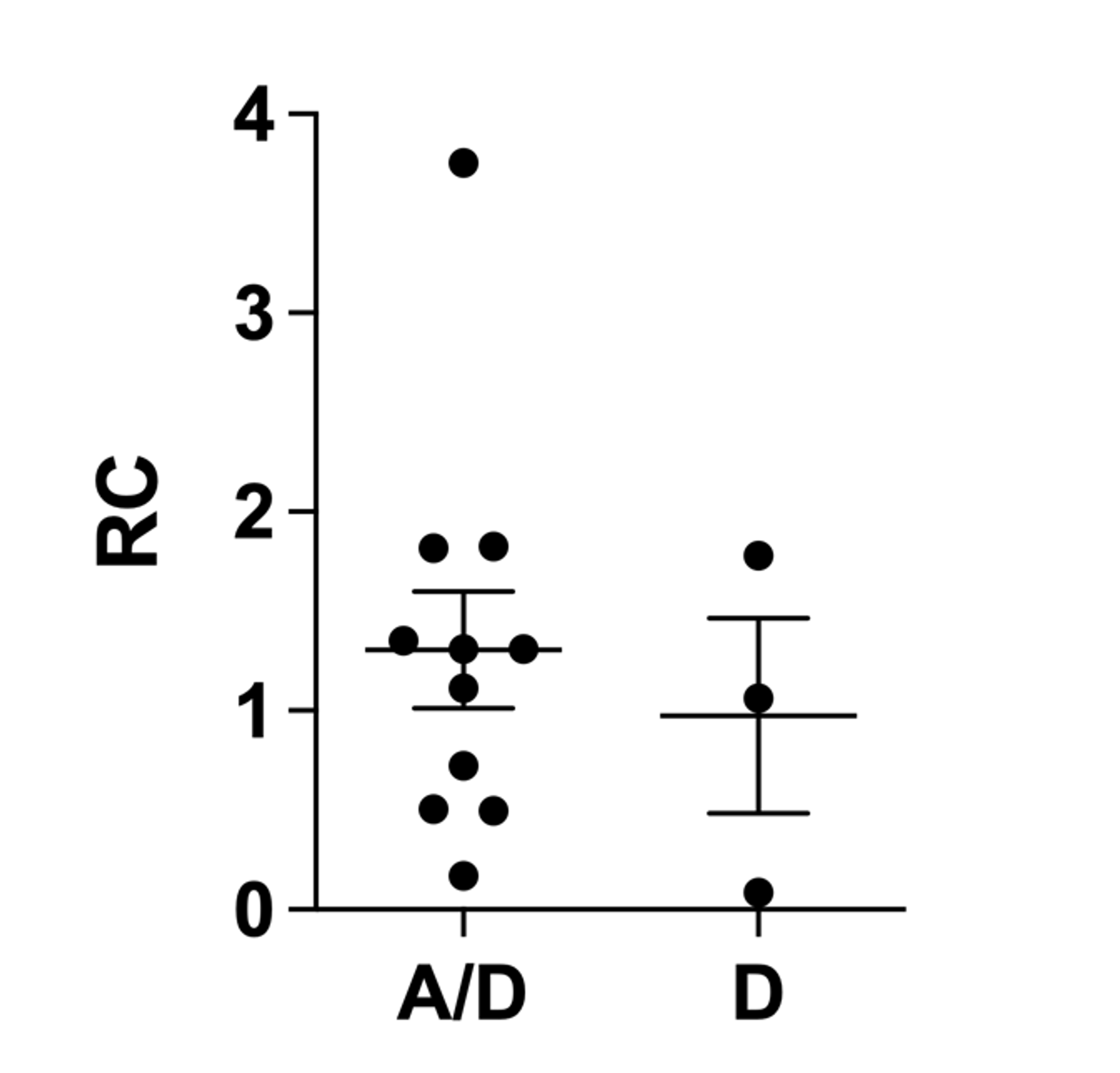

Supplement: Supplementary file 1 [file viruses-14-00334-s001.zip › viruses-1501758-supplementary/S 2.tif]
